# Distribution of axial length in Chinese congenital ectopia lentis patients: a retrospective study

**DOI:** 10.1186/s12886-017-0508-1

**Published:** 2017-07-03

**Authors:** Yichi Zhang, Guangming Jin, Qianzhong Cao, Junxiong Lin, Jianqiang Lin, Yiyao Wang, Su Ern Poh, Charlotte Aimee Young, Danying Zheng

**Affiliations:** 1 Department of Ophthalmology, Guangdong Provincial Key Laboratory of Malignant Tumor Epigenetics and Gene Regulation, Sun Yat-sen Memorial Hospital, Sun Yat-sen University, Guangzhou, China; 2 State Key Laboratory of Ophthalmology, Zhongshan Ophthalmic Center, Sun Yat-sen University, Guangzhou, China; 3 Monash School of Medicine, Monash University, Melbourne, Australia; 4 Department of Ophthalmology, University of California, San Francisco, CA USA

**Keywords:** Congenital ectopia lentis, Axial length, Refraction error, Marfan syndrome

## Abstract

**Background:**

Congenital ectopia lentis (CEL) usually leads to refractive error and may influence the axial length development. But few investigations have reported patient demographics and the distribution of axial length (AL) before surgery in Chinese pediatric patients with CEL. To describe the distribution of AL before surgery in CEL patients and its relationship with patients’ demographics, such as age, Marfan syndrome, sex, and laterality.

**Methods:**

This retrospective study reviewed 306 CEL patients from January 1, 2006 to December 31, 2015. One eye was randomly selected from each patient if both eyes were EL. The influences of Marfan syndrome, sex, and laterality to AL in different age subgroups were evaluated and compared. The differences of the AL between groups were assessed using the student t test or paired t-test. *P*-values less than 0.05 were considered statistically significant.

**Results:**

Two hundred forty-seven eyes were enrolled. 58.3% of all the patients had binoculus EL, 70% of all the patients were male and 36% of all the patients were diagnosed with Marfan syndrome. The mean AL of EL patients was 25.1 ± 2.5 mm. There was no statistical difference in the AL between patients with and without Marfan syndrome, and in the AL between male and female patients. There was statistical difference in AL between the EL-affected eye and the unaffected eye in monocular EL patients younger than 12 years old.

**Conclusions:**

This study suggests that AL can be influenced by CEL, but the influence of CEL may be reduced after the age of 12 years old, which will likely provide a useful reference when considering the most appropriate time of surgery.

## Background

Ectopia lentis (EL) is defined as displacement of the lens from its normal position. EL may be hereditary or secondary to other causes, the most common secondary cause being trauma [1]. The hereditary causes can be broadly divided into systemic disorders, such as Marfan syndrome [2], Weill-Marchesani syndrome [3], homocystinuria [4] and so on, or those without systemic disorders [1, 5, 6]. EL can also be divided into subluxated lens or luxated lens by the location of the lens. Subluxated lens refers to a partial displacement of the lens, with some of the zonules remaining intact so that part of the lens remains in the pupillary area. Luxated lens is the complete separation of all zonular attachments, so the lens completely displaced from the pupil.

EL usually leads to high refractive error [7], which can lead to defocus and form deprivation. Just as a body grows from birth through adolescence, the children’s eyes grow from infancy until adult. The refractive components in the eye grows in a proportional pattern, axial length increases dramatically in the first 2 years of life then grows at a slower rate into the second decade of life [8]. The total refraction of whole eye is balanced by the increasing axial length and decreasing power of the crystalline lens and cornea, and finally maintains at or near emmetropia throughout the entire lifetime [8]. However, the development of AL can be affected by defocus or deprivation [9]. On the other hand, the AL of patients with EL also can be influenced by genetic mutation like fibrillin-1 (FBN1) [5]. But to our knowledge, few investigations have reported patient demographics and the distribution of AL before surgery in Chinese pediatric patients with Congenital ectopia lentis (CEL). In this retrospective study, we describe the status of axial length in EL patients age ≤ 18 years old and the relationship between AL development and patient demographics, including age, sex, and laterality, and our results can provide a useful reference for the most appropriate timing for EL treatment.

## Methods

### Subjects and data collection

This research was designed as a retrospective research. It followed the Declaration of Helsinki. Local ethical approval was obtained from the ethics committee of Zhongshan Ophthalmic Center (ZOC) in Sun Yat-sen University, Guangzhou, China. The medical charts of CEL surgery patients in ZOC from January 1, 2006 to December 31, 2015 were reviewed as follow. EL is defined as displacement of the lens from its normal position. In the database of the Medical Records Department of the ZOC, CEL and ocular abnormalities are coded using the International Statistical Classification of Diseases and Related Health Problems 10th Revision (ICD-10). The research studied CEL in-patients younger than 18-years-old who were treated from January 1, 2006 to December 31, 2015, at ZOC in China. The cases with the following codes were identified in the records: congenital displaced lens (Q12.1), spherophakia (Q12.4), other congenital malformations of the anterior segment of eye (Q13.8), congenital malformation syndromes predominantly affecting facial appearance (Q87.0), Marfan syndrome (Q87.4), and dislocation of lens (H27.1). Every case record was accepted and reviewed by two independent researchers to confirm the presence of ectopia lentis in the absence of head trauma, ocular trauma, lens dislocation secondary to tumor or surgical operation. For subjects diagnosed with binocular EL, one random eye would be enrolled. For subjects diagnosed with monocular EL, the affected eye was enrolled. The age was recorded as the age of treatment. The inclusion criteria were as follow: (1) AL measurements determined by Partial Coherence Interferometry (IOLMaster, Software V5.4 and above, Carl Zeiss Meditec, Inc., Dublin, CA, USA), and the acquired AL was measured before treatment; (2) patients who were 18 years old and younger. The exclusion criteria were as follow: (1) patients with a history of previous intraocular surgery; (2) preexisting ocular diseases that may influence AL, including glaucoma, cataract or other ocular diseases, leading to defocus or deprivation; (3) patients with lens dislocation with head trauma, ocular trauma or lens dislocation secondary to tumor or surgical operation. The diagnosis of Marfan syndrome was according to the Ghent-2 criteria [10]. The patients’ AL were collected for further analysis.

### Statistical analysis

The differences of the AL between groups were assessed using the Mann-Whitney U test or Wilcoxon signed-rank test. The Bonferroni correction was used for multiple comparisons. *P*-values less than 0.05 were considered statistically significant. Statistical analyses were performed by SPSS software (version 19.0, SPSS, Inc.; Chicago, IL, USA) and Microsoft Excel (Microsoft Corporation, Redmond, Washington, USA). Means were expressed as mean ± standard deviation (SD).

## Results

### Patient demographics

**Fig. 1:**
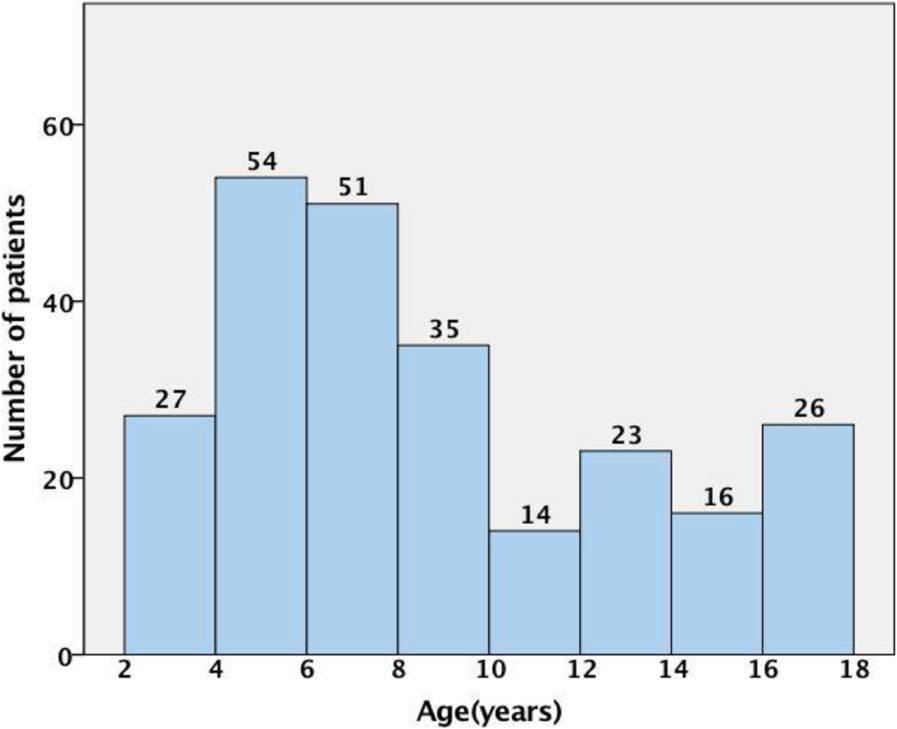
The distribution of EL patients according to age groups

The records of 306 CEL patients were reviewed, of which 247 subjects met the criteria and were enrolled in this study. All 247 patients were Chinese and their AL were collected. Table 1 summarizes the demographics of patients enrolled in this study. In our study, the patients with systemic diseases included 1 Marchesani syndrome, 1 Francois syndrome and 89 Marfan syndrome. The age group that underwent the greatest number of surgeries was patients from 4 to 6 years old (54 cases), followed by patients from 6 to 8 years old (51 cases). The number of patients from 4 to 8 years old was 105, making up 42.5% of the total EL patients (Fig. 1). Due to the character of AL growth of children, patients were divided into four age subgroups for analysis: less than 3 years old, 3 to 6 years old, 6 to 12 years old and 12 to 18 years old.

**Table 1:** Demographics of Subjects

	0–3 y (5)		3–6 y (76)		6-12y(100)		12-18y (66)		Total (247)	
	n	%	n	%	n	%	n	%	n	%
Affected eye										
Monocular	1	20.0	26	34.2	37	37.0	39	59.1	103	41.7
Binoculus	4	80.0	50	65.8	63	63.0	27	40.9	144	58.3
Sex										
Male	3	60.0	55	72.4	69	69.0	46	69.7	173	70.0
Female	2	40.0	21	27.6	31	31.0	20	30.3	74	30.0
With systemic diseases										
Yes	0	0	19	25.0	34	34.0	36	54.5	89	36.0
No	5	100.0	57	75.0	66	66.0	30	45.5	158	64.0

### The AL distribution

**Fig. 2:**
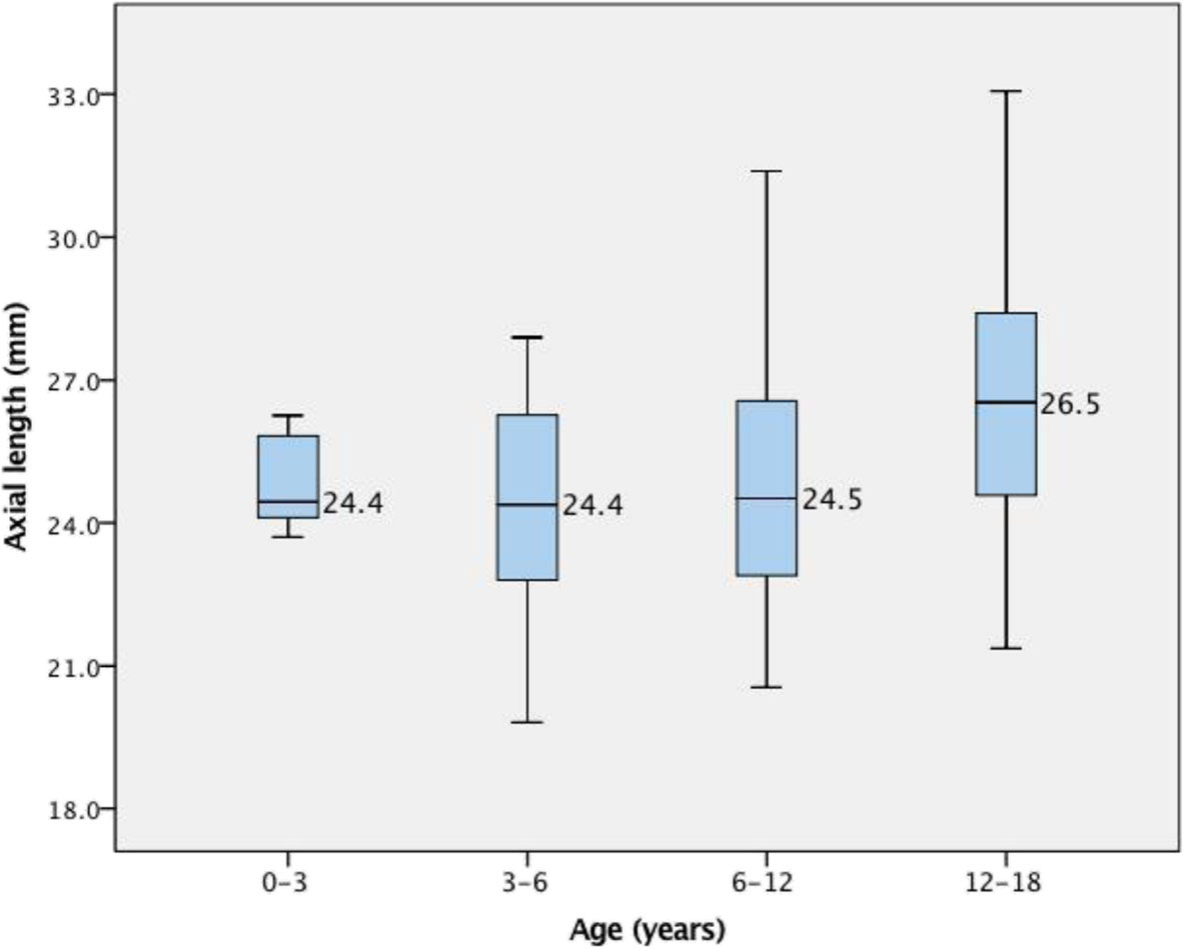
Boxplots showing the distribution and median of axial length of EL patients according to age subgroups

**Fig. 3:**
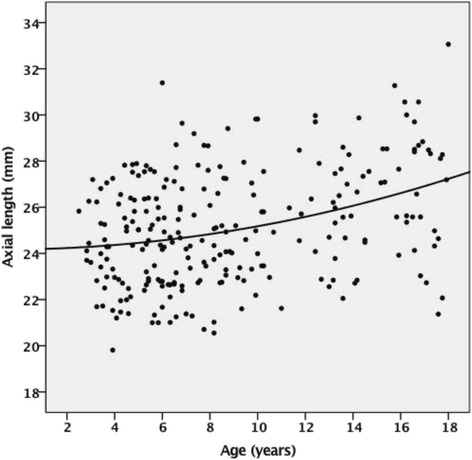
Scatterplot of axial length by patient’s age and a fitted curve of patients with EL

The AL in CEL patients from our study was found to be longer than that of normal children from other previous studies [8] (Table 2 and Fig. 2), and the median values of AL in all four subgroups were greater than 24 mm (mm). There was no statistical difference between AL of less than 3 years old, 3 to 6 years old, 6 to 12 years old subgroups, however the AL of 12 years old to 18 years old subgroup was statistically different to other subgroups (Table 2). The trend of AL distribution did not present as a logarithmic distribution (Fig. 3), which may be due to the little patients younger than two years old included in our study.

**Table 2:** Mean AL of EL patients in different age subgroup

Age subgroup	AL (mm)	n
0-3y	24.9 ± 1.1	5
3–6 y	24.4 ± 2.1	76
6–12 y	24.8 ± 2.4	100
12–18 y*	26.5 ± 2.6	66
Total	25.1 ± 2.5	247

*Abbreviations: AL = Axial Length; EL = ectopia lentis*

*p ≤ 0.008 were considered statistically significant

### Relationship between AL and different factors

To further explore the factors influencing AL growth of CEL patients, we analyzed the relationship between AL and Marfan syndrome, gender and laterality (Table 3). There was no statistical difference between AL of CEL patients with Marfan syndrome and without Marfan syndrome in all age subgroups. There was no statistical difference in the AL between male and female, except for the 12 to 18 years old subgroup. To further study the relationship of EL and AL, we compared the AL of the affected eye and unaffected eye in patients with monocular EL. The data showed that apart from the 12 years old to 18 years old subgroup which had no statistical difference between EL eye and normal eye, all the other age subgroups have statistical differences between EL eye and normal eye, and the AL of total patients also have statistical difference between EL eye and normal eye (Table 3).

**Table 3:** Comparison of the Axial Length between Different Factors by adjusting Age of EL

Subgroups	Axial length (mean ± SD) (Number of subjects)				
	0-3y	3–6 y	6–12 y	12–18 y	Total
Marfan syndrome					
With Marfan syndrome	-(0)	24.9 ± 2.1(19)	25.4 ± 2.3(34)	26.8 ± 2.4(36)	25.8 ± 2.4(89)
non-Marfan syndrome	24.8 ± 1.1(5)	24.3 ± 2.1(57)	24.5 ± 2.4(66)	26.1 ± 2.7(30)	24.7 ± 2.4(158)
*p*	-	0.58	0.95	0.53	0.69
Sex					
Male	24.8 ± 1.2(3)	24.6 ± 2.1(55)	24.9 ± 2.2(69)	27.0 ± 2.7(46)	25.4 ± 2.5(173)
Female	25.0 ± 1.3(2)	23.9 ± 2.2(21)	24.5 ± 2.7(31)	25.1 ± 1.7(20)	24.5 ± 2.3(74)
*p*	0.80	0.53	0.47	0.02*	0.26
Unilateral EL patients					
Effected eye	24.1(1)	24.4 ± 2.2(26)	24.5 ± 2.4(37)	26.8 ± 2.4(39)	25.2 ± 2.5(103)
Fellow eye	21.6(1)	24.9 ± 2.4(26)	25.1 ± 2.4(37)	26.5 ± 2.2(39)	25.7 ± 2.5(103)
*p*	-	0.01*	0.00*	0.65	0.00*

*Abbreviations: EL = ectopia lentis*

*p ≤ 0.05 were considered statistically significant

## Discussion

AL is a crucial parameter of eyeball development and can be influenced by many factors such as genetics and environment [1, 6]. The environment influence to development of AL may be varied in different ages [9]. Thus, to describe the distribution AL tendency before surgery can be a helpful reference for estimating the most appropriate time of surgery. However, the published studies on AL development in CEL patients are scant currently. As we know, this study had the largest sample to describe the AL distribution in CEL patients and its relationship between patients’ demographics, including age, gender, laterality and Marfan syndrome.

In this study, 58.3% of enrolled patients had Binocular EL, 70% of enrolled patients were male and 36% of enrolled patients were diagnosed with Marfan syndrome. Furthermore, 42.5% of total patients were in age subgroup four to eight years old. Some possible explanations for this is that children younger than age four may have difficulty expressing themselves well or their patients did not notice the abnormal eye appeared with EL, thus delaying the time of first diagnosis and treatment of EL at the hospital. Another possibility is that the onset of CEL may usually between age 4 and 8. However, there are few studies that have addressed this issue, hence it may require larger and prospective study to explain this phenomenon.

As the body, the children’s eyeball grows from birth to adulthood. The refractive components grow in a proportional pattern: In the first two years, AL increases dramatically, then it grows at a slower rate until adulthood. The steep cornea in infancy becomes flat in the first 18 months, then almost does not change in the rest of life. The power of the lens also declines and its curvature decreases as age grows in childhood. Matched by the development of the lens and cornea, the AL length results in declining optical power. Thus, whole eye refraction is balanced by the increasing AL and decreasing power of the lens and cornea, and finally equilibrated at or near emmetropia throughout the entire lifetime [8]. However, when an infant or a child has EL, the absence of lens will result in refraction error or vision deprivation. This may cause the AL to lengthen faster [9], resulting in myopia or even high myopia. Our study revealed that the mean AL in CEL eyes is longer than that in normal eyes [8], which is consistent with previous studies [11–15].

To explore the factors influencing AL in CEL patients, the relationship between AL and Marfan syndrome, gender and laterality was further analyzed. Marfan syndrome is an autosomal dominant connective tissue disorder affecting several systems [16]. It is usually caused by FBN1 mutations, encoding the connective tissue protein fibrillin-1 [17]. EL was found to be a part of Marfan syndrome [16]. In 2010, the Ghent-2 criteria was presented. In these diagnostic criteria, aortic root-aneurysm/dissection and EL are the crucial features of Marfan syndrome [10]. The previous studies showed that 37% to 87% Marfan syndrome patients had EL [13, 18–20], with other ocular characters in Marfan syndrome bilaterally and symmetrically, including flatted corneas and increased AL [14]. Previous study suggested that expression of abnormal fibrillin in Marfan syndrome may lead to enlargement of the eyeball, which may be the cause of a longer AL in Marfan syndrome [5]. However, in our study, the AL was compared between patients with Marfan syndrome and patients without Marfan syndrome, and it was found that there was no statistical difference between them. It is possible that the longer AL of EL eye in Marfan syndrome patient may be mainly caused by the defocus or deprivation, and minor influence of the gene. Another explanation is that the non-Marfan syndrome patients may also have mutation in FBN1 gene or other gene resulted in longer AL [6]. Thus, more prospective studies should be designed to verify this hypothesis.

Previous studies have suggested that sex-linked differences are found in the infants and children’s AL. Compared with female infants, the ALs grow faster in male infants. The mean AL is shorter in girls than in boys [21–23]. In the present study, it is found that the ALs was significantly longer in male than in female in 12–18 years old age subgroup. One possible explanation for this result is that the boys with EL may have endured EL for a longer time, making it harder for their parents to be aware or to detect the abnormality, which delayed the time of treatment. It is also possible that the relatively small number of subjects can lead to statistical biases.

Furthermore, the impact of EL on the development of AL by laterality was analyzed. A longer AL was noted in the affected eyes of patients with unilateral EL in 0–3 years old subgroup, 3–6 years old subgroup, 6–12 years old subgroup. But no statistical difference was found in 12–18 years old subgroup. One explanation is that the influence of EL to the development of AL may be significant in children younger than 12 years old. As the patient is older, the development of axial length is almost done, and the influence of EL is minimal.

Our results suggest that the development of AL may be more seriously affected by CEL in patients younger than 12 years old. Primary IOL implantation in these patients could cause future myopic shift, greater prediction error and unmatched IOL size, and so on. On the other hand, the tightness of the suture and the position of scleral-fixated intraocular lens may vary with the change of AL and cause some unexpected of postoperative complications. This implies that CEL patients younger than 12 years old may need intensive follow-up and the treatment strategies for these patients desire more considering.

This study results must be assessed within its context limitation. Firstly, this study is a single-center study, which cannot represent the whole population. It only studied the surgical patients in the Zhongshan Ophthalmic Eye Centre, thus may involve biases, as patients who did not received treatment in the hospital were excluded from the study. Secondly, most of our patients cannot provide the exact duration of EL. Their parents discovered the EL, when EL is obviously or the patients complained can see double image. This may cause some bias in the results when analyzed the influences of Marfan syndrome and gender. Thirdly, the absence of refraction data in this study limited the analysis on refraction influence in the development of AL in CEL patients. Despite these limitations, this study has its strengths, as it was conducted with a large sample size, and it described the distribution of AL before surgery in Chinese CEL patients, which would be a useful reference for judging the timing of surgery.

## Conclusion

In conclusion, this study suggests that CEL can increased the AL of patients, but the influence may be reduced after the age of 12 years old, which will likely provide a useful reference when considering the most appropriate time for surgery.

## Abbreviations

AL: axial length
CEL: congenital ectopia lentis
EL: ectopia lentis
FBN1: genetic mutation like fibrillin-1
ICD-10: the International Statistical Classification of Diseases and Related Health Problems 10th Revision
ZOC: Zhongshan Ophthalmic Center

## References

[1] Neely DE, Plager DA (2001). Management of ectopia lentis in children. Ophthalmol Clin N Am.

[2] Castellano JM, Silvay G, Castillo JG (2014). Marfan syndrome: clinical, surgical, and anesthetic considerations. Semin Cardiothorac Vasc Anesth.

[3] Fujiwara H, Takigawa Y, Ueno S, Okuda K (1990). Histology of the lens in the Weill-Marchesani syndrome. Br J Ophthalmol.

[4] Abbott MH, Hussels IE. Ectopia lentis due to homocystinuria. Birth Defects Orig Artic Ser. 1971;7(3):170–2. 5173135

[5] Ades LC, Holman KJ, Brett MS, Edwards MJ, Bennetts B. Ectopia lentis phenotypes and the FBN1 gene. Am J Med Genet A. 2004;126a(3):284–289. 10.1002/ajmg.a.20605 15054843

[6] Sadiq MA, Vanderveen D (2013). Genetics of ectopia lentis. Semin Ophthalmol.

[7] Rasooly R, Benezra D. Unilateral lens dislocation and axial elongation in Marfan syndrome. Ophthalmic Paediatr Genet. 1988;9(2):135–6. 10.3109/13816818809031488 3263606

[8] Gordon RA, Donzis PB. Refractive development of the human eye. Arch Ophthalmol. (Chicago, Ill : 1960). 1985;103(6):785–789. 10.1001/archopht.1985.01050060045020 4004614

[9] McClatchey SK, Dahan E, Maselli E, Gimbel HV, Wilson ME, Lambert SR, Buckley EG, Freedman SF, Plager DA, Parks MM (2000). A comparison of the rate of refractive growth in pediatric aphakic and pseudophakic eyes. Ophthalmology.

[10] Loeys BL, Dietz HC, Braverman AC, Callewaert BL, De Backer J, Devereux RB, Hilhorst-Hofstee Y, Jondeau G, Faivre L, Milewicz DM (2010). The revised Ghent nosology for the Marfan syndrome. J Med Genet.

[11] Park SC, Chung ES, Chung TY, Kim SA, Oh SY (2010). Axial growth and binocular function following bilateral lensectomy and scleral fixation of an intraocular lens in nontraumatic ectopia lentis. Jpn J Ophthalmol.

[12] Chandra A, Aragon-Martin JA, Hughes K, Gati S, Reddy MA, Deshpande C, Cormack G, Child AH, Charteris DG, Arno G (2012). A genotype-phenotype comparison of ADAMTSL4 and FBN1 in isolated ectopia lentis. Invest Ophthalmol Vis Sci.

[13] Konradsen TR, Zetterstrom C (2013). A descriptive study of ocular characteristics in Marfan syndrome. Acta Ophthalmol.

[14] Drolsum L, Rand-Hendriksen S, Paus B, Geiran OR, Semb SO. Ocular findings in 87 adults with Ghent-1 verified Marfan syndrome. Acta Ophthalmol. 2015;93(1):46–53. 10.1111/aos.12448 24853997

[15] Evereklioglu C, Hepsen IF, Er H. Weill-Marchesani syndrome in three generations. Eye (Lond). 1999;13(Pt 6):773–777. 10.1038/eye.1999.226 10707143

[16] Henschen BL, Bierman JA, Wayne DB, Ryan ER, Thomas JX, Curry RH, Evans DB. Four-Year Educational and Patient Care Outcomes of a Team-Based Primary Care Longitudinal Clerkship. Acad Med: J Assoc Am Med Coll. 2015;90(11 Suppl):S43–49. 10.1097/ACM.0000000000000897 26505100

[17] De Paepe A, Devereux RB, Dietz HC, Hennekam RC, Pyeritz RE (1996). Revised diagnostic criteria for the Marfan syndrome. Am j Med Genet.

[18] Arbustini E, Grasso M, Ansaldi S, Malattia C, Pilotto A, Porcu E, Disabella E, Marziliano N, Pisani A, Lanzarini L, et al. Identification of sixty-two novel and twelve known FBN1 mutations in eighty-one unrelated probands with Marfan syndrome and other fibrillinopathies. Hum Mutat. 2005;26(5):494. 10.1002/humu.9377 16222657

[19] Rybczynski M, Bernhardt AM, Rehder U, Fuisting B, Meiss L, Voss U, Habermann C, Detter C, Robinson PN, Arslan-Kirchner M et al. The spectrum of syndromes and manifestations in individuals screened for suspected Marfan syndrome. Am J Med Genet Part A. 2008;146a(24):3157–3166. 10.1002/ajmg.a.32595 19012347

[20] Kara N, Bozkurt E, Baz O, Altinkaynak H, Dundar H, Yuksel K, Yazici AT, Demirok A, Candan S (2012). Corneal biomechanical properties and intraocular pressure measurement in Marfan patients. J Cataract Refract Surg.

[21] Larsen JS (1971). The sagittal growth of the eye. 3. Ultrasonic measurement of the posterior segment (axial length of the vitreous) from birth to puberty. Acta Ophthalmol (Copenh).

[22] Isenberg SJ, Neumann D, Cheong PY, Ling YL, McCall LC, Ziffer AJ. Growth of the internal and external eye in term and preterm infants. Ophthalmol. 1995;102(5):827–30. 10.1016/s0161-6420(95)30950-5 7777283

[23] Trivedi RH, Wilson ME (2007). Biometry data from caucasian and african-american cataractous pediatric eyes. Invest Ophthalmol Vis Sci.

